# Addiction as Learned Behavior Patterns

**DOI:** 10.3390/jcm8081086

**Published:** 2019-07-24

**Authors:** Andreas Heinz, Anne Beck, Melissa Gül Halil, Maximilian Pilhatsch, Michael N. Smolka, Shuyan Liu

**Affiliations:** 1 Department of Psychiatry and Psychotherapy, Charité–Universitätsmedizin Berlin (Campus Charité Mitte), 10117 Berlin, Germany; 2 Department of Psychiatry and Psychotherapy, Technische Universität Dresden, 01187 Dresden, Germany; 3 Neuroimaging Center, Technische Universität Dresden, 01187 Dresden, Germany

**Keywords:** substance use disorders, alternative reward, cue exposure, animal and computational models, behavioral control, craving and relapse, habit formation

## Abstract

Individuals with substance use disorders (SUDs) have to cope with drug-related cues and contexts which can affect instrumental drug seeking, as shown with Pavlovian-to-instrumental transfer (PIT) tasks among humans and animals. Our review addresses two potential mechanisms that may contribute to habitual or even compulsive drug seeking and taking. One mechanism is represented by Pavlovian and PIT effects on drug intake. The other is a shift from goal-directed to habitual drug intake, which can be accessed via model-based versus model-free decision-making in respective learning tasks. We discuss the impact of these learning mechanisms on drug consumption. First, we describe how Pavlovian and instrumental learning mechanisms interact in drug addiction. Secondly, we address the effects of acute and chronic stress exposure on behavioral and neural PIT effects in alcohol use disorder (AUD). Thirdly, we discuss how these learning mechanisms and their respective neurobiological correlates can contribute to losing versus regaining control over drug intake. Utilizing mobile technology (mobile applications on smartphones including games that measure learning mechanisms, activity bracelets), computational models, and real-world data may help to better identify patients with a high relapse risk and to offer targeted behavioral and pharmacotherapeutic interventions for vulnerable patients.

## 1. Introduction

Drugs of abuse stimulate dopamine release and thus reinforce drug intake [1]. Wise originally suggested that dopamine release is tied to pleasure and hedonic changes that strongly reinforce the behavior of repetitive drug use [2]. Robinson and Berridge later suggested that dopamine release is more associated with reward motivation rather than mediating hedonic pleasure, contributing to “wanting” or “craving” instead of “liking” drugs of abuse [3]. This hypothesis was based on studies by Schulz and co-workers [4]. They found that phasic dopamine release is modulated by an unexpected reward and a conditioned stimulus, which in turn reliably predict reward. They suggested dopamine signals code reward prediction errors (i.e., the difference between received and predicted rewards) which drive reward-motivated behaviors. Accordingly, dopamine D2-receptor blockade in humans was associated with motivational deficits, but not anhedonia [5]. Based on the observations above, dopamine is not only associated with the encoding of unexpected rewards, but also the attribution of incentive salience to reward-related cues [3]. Further research is required to better understand how such learning mechanisms may shed light on drug seeking and intake. Specifically, recreational drug use elicits a rather strong dopamine release, thus reinforcing drug consumption [6]. Habitual drug use is characterized by a shift from ventral to dorsal striatal processing, including the dopaminergic modulation in fronto-striatal brain circuitries [6]. Ultimately, drug consumption was independent of rewarding or aversive outcomes [6,7]. The pathways from the orbitofrontal cortex to the dorsal striatum play a key role in compulsive drug use, in spite of aversive consequences [8]. Obsessions and compulsions in obsessive compulsive disorder (OCD) differ from drug craving and intake [9]. However, drug addiction is characterized by compulsive drug intake and has substantial similarities with other disorders of compulsions, including OCD, on phenomenological and neurobiological levels [10,11]. In this review, we discuss two potential mechanisms that may contribute to habitual drug intake and, ultimately, drug seeking and taking. One mechanism is the stimulus response associations as represented by Pavlovian effects on drug intake and the other is a shift from goal-directed to habitual drug intake, which can be accessed via model-based versus model-free decision-making in respective learning tasks [11,12].

## 2. Pavlovian Mechanisms in Addictive Behavior

Drug-associated cues can elicit drug craving and promote drug seeking [3,13]. From a theoretical point of view, Pavlovian unconditioned cues, such as food, elicit unconditioned responses, including increased salivation and food craving. Conditioned cues, such as pictures of alcoholic beverages, may elicit drug craving as a conditioned response [14]. However, most drugs of abuse do not often come accidentally to an addicted person. Instead, patients with drug dependence actively search for available drugs. One of our patients described the situation with the following words: “When the evening comes and the sky turns grey, I pass by these bars with their warm yellow light and hear the clinging of glasses. I’m lost.” In this context, conditioned cues include the clinging of glasses, certain colors of light in a bar, and the kind of loneliness while looking at the dark gloomy sky. These conditioned cues have been previously paired with positive/pleasant activities/evenings. Such conditioned contextual cues elicit drug craving and have an impact on goal-directed behavior; the afflicted person changes his or her direction, enters the bar, orders a drink, and consumes it. The implicated mechanism has been called Pavlovian-to-instrumental transfer (PIT) [15]. During PIT, a Pavlovian conditioned cue (e.g., the clinging of glasses) can have an impact on a series of obviously unrelated approach behavioral sequences, including entering a certain place, talking to bartenders, and ordering a drink. Regarding cue reactivity, imaging studies show that functional activation elicited by drug-associated cues, particularly in the medial prefrontal cortex, was correlated with a high risk of relapse for detoxified patients with alcohol use disorder (AUD) [16,17]. Moreover, naltrexone, which blocks μ-opioid receptors that have been reported to be elevated in AUD, also reduces cue-induced functional activation in the ventral striatum in AUD patients [18,19]. Another neurotransmitter system implicated in cue-induced brain activation in addictive disorders is the dopamine system. A low availability of dopamine D2-receptors in the ventral striatum is associated with increased functional activation elicited by alcohol cues in the medial prefrontal cortex [20]. Low dopamine D2-receptor availability following detoxification may represent a counter-regulatory new adaptation following excessive dopamine release due to the consumption of drugs of abuse and delayed recovery of dopamine D2-receptor sensitivity following detoxification was associated with poor treatment outcomes [21].

**Figure 1:**
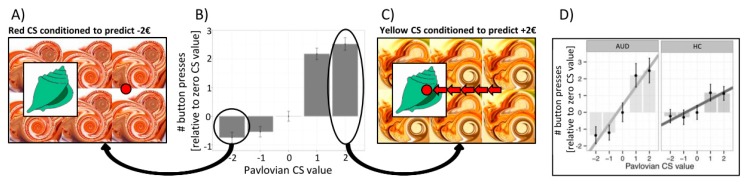
The Pavlovian-to-instrumental (PIT) effect. (**A**) The unrelated Pavlovian stimulus (conditioned stimulus (CS)) presented in the background is negatively valued because it has previously been paired with passive monetary loss. (**B**) The PIT effect is indicated by the number of button presses (instrumental response) as a function of the value of the respective Pavlovian background stimulus (−€2, −€1, €0, +€1, +€2). (**C**) Combining the shell with a positive Pavlovian cue in the background of the screen increases approach behavior (number of button presses) in the unrelated instrumental task. (**D**) The PIT effect was significantly stronger in subjects with alcohol use disorder (AUD) compared to healthy controls (HC).

So how can alcohol cues trigger not only drug craving and functional activation in the ventral striatum, amygdala, and medial prefrontal cortex [17,22], but also bias complex goal-directed behavior toward drug seeking and intake? A subclass of environmental cues is called Pavlovian conditioned stimuli due to the ability to elicit a conditioned response, which is usually inborn (such as the production of saliva in a hungry dog or avoidance of malodors) and hence hard-wired in the central nervous system [23]. As suggested above, such Pavlovian conditioned stimuli can also impact ongoing instrumental behavior, even if the instrumental behavior was acquired independently of Pavlovian conditioning, a process called Pavlovian-to-instrumental transfer (PIT) [24]. In PIT, positively valued Pavlovian cues promote instrumental responses and approach behaviors (e.g., enhance the frequency of pressing a button) [24], while negatively valued Pavlovian cues promote inhibition or withdrawal actions (e.g., lower the frequency of pressing a button for instrumental approach or enhance the frequency of pressing a button for instrumental withdrawal [25] (Figure 1)). Thus, in drug addiction, Pavlovian conditioned cues can bias instrumental behavior toward drug seeking and intake [26,27,28].

In outcome-specific PIT, presenting a particular reward-predicting cue can selectively elevate instrumental responses that are associated with the same unique reward, while in general PIT, a reward- or loss-predicting cue can generally modify instrumental responses toward any outcome [24]. So-called single-lever PIT tasks (see Figure 1) usually reflect general PIT, while a full transfer task enables the disentanglement between general and outcome-specific PIT [24]. Like habits, PIT effects may help to prune a complex “decision tree” by biasing an individual to instrumental approaches or withdrawal behaviors in the presence of certain background stimuli [29]. Indeed, a general tendency to rely on habitual rather than complex goal-directed decision-making was associated with increased PIT effects in healthy volunteers [30]. Moreover, we observed PIT effects being modulated by personality traits, such as impulsive decision making, with the strongest PIT effects observed in high impulsive alcohol-dependent patients compared to low impulsive patients [31].

PIT effects may be specifically strong in stressful situations, when decisions have to be fast, and profit from an overall “atmospheric” evaluation of the dangerousness or safety of the current situation [32]. Various forms of stress promote substance use and relapse, as evidenced by a broad range of literature [33,34]. In this context, Quail and co-workers suggested that stress exposure modifies the influence of Pavlovian cues on behavior [35]. They observed that subjects reporting high stress were impaired to suppress instrumental responding under no-reward Pavlovian cues [35]. Moreover, acute stress selectively increased cue-triggered wanting independently of hedonic properties of the reward [36]. Stress exposure and long-term endocrine stress measures (e.g., hair cortisol) in addicts have so far not been studied with respect to PIT and its association with losing versus regaining control over drug intake. Moreover, we did not find gender and age effects [31,37,38], which would require further research.

With respect to neurobiological correlates, animal experiments and human studies suggest that activation of the basolateral amygdala, the nucleus accumbens shell, and the ventrolateral putamen contribute to an outcome-specific form of PIT [15,39,40]. The central nucleus of the amygdala and the nucleus accumbens core are involved in the general form of PIT [15,39,40]. These neurobiological differences are in line with a goal-directed aspect of specific PIT compared to an arousing effect of general PIT. In the outcome-specific form of PIT, the Pavlovian cue has been conditioned with the same rewarding outcome that can also be gained when performing the instrumental response. For example, the smell of wine promotes ordering and consuming a glass of wine instead of lemonade. In the general form of PIT, the Pavlovian cue has been conditioned to a positive outcome that is not associated with the outcome available by the instrumental action. For example, upbeat music played in a shopping mall motivates customers to spent more money. Thus, general PIT appears to promote instrumental actions by modulating arousal, while outcome-specific PIT may facilitate the retrieval of particular actions based on their outcomes [26].

In line with this, stronger general PIT effects elicited by positive non-drug cues and functional PIT-related brain activation in the nucleus accumbens were observed in prospective AUD relapsers [37,41]. This phenomenon of increased PIT effects was also observed in studies when animals were pretreated with drugs of abuse [24].

In smokers, tobacco-related PIT effects have been demonstrated in several studies in satiated and deprived smokers [42,43], but contrary to our findings in AUD patients, studies in smokers did not see stronger PIT effects in more dependent subjects or compared to non-dependent controls. In cocaine addicts, cocaine-paired cues can provoke the pursuit of cocaine through a Pavlovian motivational process [27]. In general, there are a limited numbers of studies examining whether different types of drug abuse, such as opioids and amphetamine, can support PIT [24]. Establishing these effects may deepen our understanding of the behavioral and neural processes underlying cue-motivated drug-seeking behavior.

The PIT effects of drug-related cues were also studied in subjects with AUD. Regarding alcohol versus water cues, we expected that alcohol cues would promote approach behaviors and predict poor treatment outcomes, as was the case with general PIT effects. The appetitive and aversive Pavlovian cues were passively conditioned with monetary reward or loss. Surprisingly, however, patients with poor treatment outcomes behaved similar to the healthy controls. Patients with good treatment outcomes who did not relapse in the follow-up period of three months showed a significant difference both in behavior and in functional brain responses to alcohol cues in a general PIT task [38]. They showed both an increased functional activation of the ventral striatum when confronted with these Pavlovian-conditioned alcohol cues, as well as an inhibition of approached behavior and increased withdrawal behavior in the presence of such alcohol cues [38]. Interestingly, alcohol-dependent patients with good treatment outcomes appeared to learn a specific inhibitory reaction to alcohol cues. At least, they significantly differed both from healthy controls and patients who later relapsed during the follow-up period. Increased activation of the ventral striatum may be due to salience attribution to alcohol cues, which apparently did not simply trigger approach behaviors, but instead enabled subjects to inhibit unrelated goal-directed behaviors. Thus, patients with good treatment outcomes could use alcohol cues as warning signs and—unlike the patient in the example explained above—resist drug-approach tendencies. For example, they may not enter the bar with the warm yellow light or avoid going to the supermarket where they used to buy their alcoholic beverages.

Patients may learn to use environmental cues as warning-signs and thus train to avoid rather than approach situations in which drugs are available. One training program targeting such drug-approach tendencies is the so-called Zooming Joystick Task. Patients with addictive disorders learn to push pictures of alcohol beverages away instead of pulling them toward themselves. Four training sessions appear to be sufficient to successfully reduce the relapse-risk during an one year follow-up period, with the number needed to treat (NNT) being around 10, suggesting that 10% of all patients would benefit from this intervention [44]. From a neurobiological perspective, such alcohol cues activate the medial prefrontal cortex and further brain areas, including the amygdala, implicated in PIT mechanisms; successfully learning to push alcohol cues away was associated with reduced amygdala activation in AUD patients [45,46]. The success of such training programs encourages studies to better understand the neurobiological correlates and to identify patients who may respond particularly well to such training programs.

**Figure 2:**
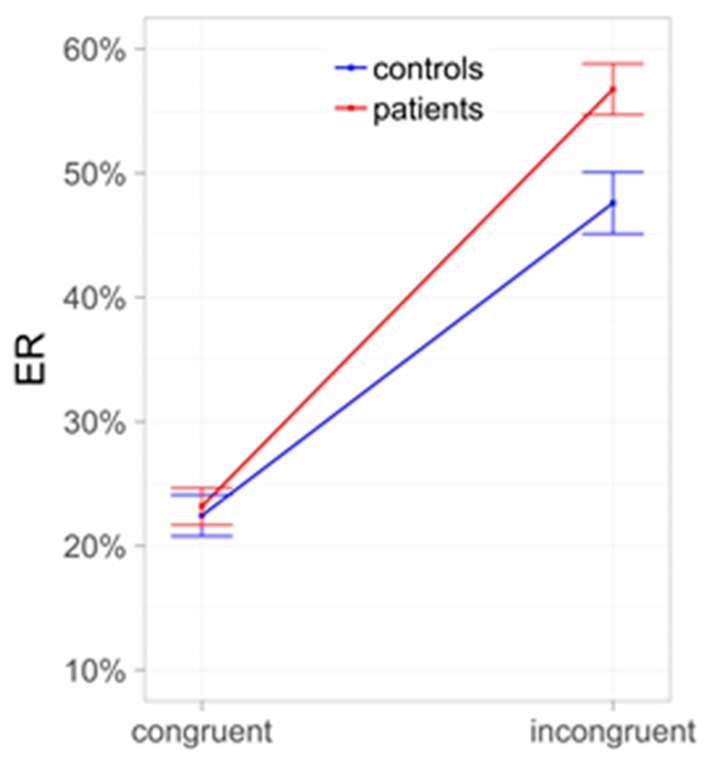
Conflict between Pavlovian and instrumental control: Subjects with alcohol use disorder (AUD) compared to controls. ER = error rates.

In line with the key role of the amygdala and nucleus accumbens, behavioral PIT effects are understood as driven by bottom-up processes. Nevertheless, a conflict—like in a Stroop task—should be elicited in situations in which Pavlovian and instrumental cues are incongruent (i.e., collecting “good” shells when negatively valued context stimuli are shown, or leaving “bad” shells during presentation of positively valued contexts) and this conflict should trigger the allocation of top-down control. Indeed, the results of Sommer and co-workers [31] revealed that instrumental behavior during PIT is more error-prone when instrumental and Pavlovian cues are incongruent, in line with the assumption of such a conflict between Pavlovian and instrumental control (Figure 2). Importantly, the incongruence effect was more pronounced in AUD subjects than in controls, indicating that reduced interference control may impair goal-directed behavior, especially in AUD subjects.

## 3. From Goal-Directed to Habitual Drug Seeking—The Importance of Contextual Cues

Dual-process theories of learning and addiction propose that the development of drug addiction involves a shift from goal-directed to habitual control of action [6,7]. Animal models of drug addiction suggest that occasional drug use becomes habitual and ultimately compulsive (i.e., it is maintained in spite of aversive consequences) [7]. In humans, complex model-based behavior is reduced in patients with different substance use disorders (SUDs) as well as with OCD [11]. This may help to explain why aversive outcomes associated with drug consumption do not affect the respective behavior and enforce modification. Regarding patients with AUD, the results of the recent studies were inconsistent. In a study by Voon and co-workers [11], a shift was not observed from model-based toward model-free behavior in AUD patients, while such a shift was observed by Sebold and co-workers [47]. However, Sebold and co-workers did not replicate their previous findings in a larger independent sample [48]. There was no overall reduction in model-based behavior in patients with AUDs and in patients with poor treatment outcomes compared to patients with good treatment outcomes [48].

Model-based versus model-free behavior and goal-directed versus habitual behavior are assessed by different tasks. Model-based versus model-free behavior is assessed via taking complex decision-making processes into account, while goal-directed versus habitual behavior is operationalized via the impact of reward devaluation. Nevertheless, both tasks are intercorrelated in the sense that individuals who tend to behave in a model-based way also show stronger goal-directed behaviors, while individuals who tend to respond in a habitual way rely more strongly on model-free decision-making [49]. Therefore, failure to observe effects of a reduction in model-based behavior in AUD patients may challenge the assumption that these patients have a general tendency for habit-formation at the expense of goal-directed decision-making. However, Sebold and co-workers also observed that model-based versus model-free behavior can predict treatment outcomes when taking alcohol expectancy into account [48]. Patients with high alcohol expectancies showing low model-based behavior, thus shifting the balance toward model-free behavior, had poor treatment outcomes [48]. These findings suggest that shifts from goal-directed to habitual decision-making depend on contextual stimuli. It may be specifically relevant for a subset of behavior patterns associated with drug seeking and drug consumption. Instead of searching for general tendencies to form habits, specific context-dependent learning mechanisms that may interfere with cognitive control and conscious decisions to remain abstinent must be identified. Cognitive abilities such as working memory have been discovered to interplay between these two behavioral systems [50,51]. Acute [52] or chronic [53] stress are thought to impair executive resources underlying working memory and were found to impair goal-directed decision-making, inducing a relative shift toward habitual behavioral control. Stress is also an important factor in the development and maintenance of AUD and has been shown to increase alcohol intake [54,55,56]. Human imaging studies revealed that acute stress enhanced stimulus–response learning, which was accompanied by increased amygdala activity during a spatial learning task [57], as well as biased choices for immediately rewarding food stimuli and increased functional connectivity between the ventromedial prefrontal cortex and amygdala and striatal regions encoding tastiness [58]. Therefore, the acute stress experience might promote loss of control over alcohol intake by diminishing goal-directed responses and promoting habitual actions, thus undermining the goal to stay abstinent by promoting habitual substance intake. We also observed that goal-directed decision-making was affected by increased life stressors [59], underlining the strong potential of interventions aimed at altering stress-related effects on losing and regaining control over substance use. In future studies, researchers could model learning and cognitive control systems in interaction with real-life monitoring of stressors, cue responsivity, and ecological momentary assessment of alcohol consumption.

## 4. Summary and Outlook

Human behavior is more flexible and dependent on context than previously assumed in straight-forward models (i.e., increased PIT effects and habitual decision-making in drug addiction). Researchers should consider contextual cues, such as expectancies and availabilities, mood states, individual stress-levels, and cognitive control processes. Modern technology allows ambulatory assessments, including reports of mood-states, recordings of geolocation, and psychomotor activity in real life [60]. An important future focus should be on the development and establishment of computational models for learning and decision-making in humans. To date, cue exposure in general has limited effects and individual differences in cue effects, including ambulatory assessments of learning mechanisms like PIT, may help to target those patients [61,62]. Thus, utilizing a model’s predictions and real-world data may help to better identify patients with a high relapse risk and to offer specific behavioral or pharmacological interventions for vulnerable patients.
